# Scientific evidence does not support oyster farming as a marine carbon dioxide removal strategy for climate mitigation

**DOI:** 10.1073/pnas.2533459123

**Published:** 2026-02-27

**Authors:** Fabrice Pernet, Philip W. Boyd, Sam Dupont, Jean-Pierre Gattuso, Frédéric Gazeau, Christopher J. Gobler, Marc Metian, Stephen Tomasetti, Phillip Williamson

**Affiliations:** ^a^Ifremer, Université de Brest, Centre National de la Recherche Scientifique, Institut de Recherche pour le Développement, Laboratoire de l'Environnement Marin, Plouzané 29280, France; ^b^Institute for Marine and Antarctic Studies, University of Tasmania, Hobart, TAS 7001, Australia; ^c^Department of Biological and Environmental Sciences, University of Gothenburg, Fiskebäckskil 451 78, Sweden and Marine and Freshwater Research Institute, Hafnarfjörður 220, Iceland; ^d^Centre National de la Recherche Scientifique, Sorbonne Université, Villefranche-sur-Mer, France; ^e^Institute for Sustainable Development and International Relations, Paris 75006, France; ^f^School of Marine and Atmospheric Sciences, Stony Brook University, Stony Brook, NY 11794-5000; ^g^International Atomic Energy Agency–Marine Environment Laboratories, Laboratories, Department of Nuclear Sciences and Applications, Principality of Monaco 98000, Monaco; ^h^Department of Natural Sciences, University of Maryland Eastern Shore, Princess Anne, MD 21853; ^i^School of Environmental Sciences, University of East Anglia, Norwich NR4 7TJ, United Kingdom

Chen et al. (1) investigated the role of oysters in the carbon cycle using mesocosms deployed in a pond. They showed that oyster suspension feeding and biodeposition may, at low stocking densities, increase the production of organic carbon in the water column, with an apparent accumulation in the sediment. The authors conclude that oyster farming is a scalable solution for marine carbon dioxide removal (mCDR).

While their study addresses an important topic, its restricted timing and duration, poor representation of oyster farming conditions, as well as biases in interpretation and unsupported claims, prevent it from supporting such conclusions.

Regarding the timing and duration of the study, three points should be noted. First, measurements were conducted only between June and October, when conditions are favorable for photosynthesis, overestimating annually integrated CO_2_ uptake. Full seasonal coverage, including winter periods when conditions are more heterotrophic, is needed to provide a reliable estimate of the carbon budget (2, 3).

Second, Chen et al. do not provide a whole carbon budget for oyster farming. Key processes likely to cause significant CO_2_ emissions are spat production, harvest, transport, depuration, consumption of oyster flesh, and the disposal of shells which are frequently incinerated (4, 5). All these stages need to be covered in a comprehensive life cycle analysis (LCA) that is specific to their aquaculture system. The two LCA studies cited in the paper are of limited relevance, since they relate to clams and mussels, and their findings are not supported by other work (2, 6).

Third, shallow, short-term sedimentation of organic carbon was considered a carbon sink despite its extreme susceptibility to remineralization over longer periods. Given that climate-mitigation strategies require carbon sequestration on multidecadal, preferably geological, timescales (7), realistic remineralization needs to be incorporated into the carbon budget.

There are key differences between the closed mesocosms and open field conditions for oyster farming that limit the generalization of the study. In particular, oyster stocking densities were very low [0.5 to 4 ind m^−2^ compared to 80 to 300 ind m^−2^ in typical oyster aquaculture (8)] and the highest stocking density used did not show any enhancement of primary production relative to the control without oysters. Furthermore, nutrients were added to stimulate phytoplankton growth but nutrient concentrations compared to natural levels were not reported. Finally, oxygen exchange was likely limited in these closed mesocosms held in a closed pond, likely to favor carbon storage due to low oxygen concentration in the sediment.

The field data do not support the claim that aquaculture enhances photosynthesis and, thus, carbon uptake. Instead, the chlorophyll and oxygen measurements seem to indicate the opposite effect. Furthermore, the data and chosen analyses were inadequate to support any assertion that waters surrounding the farms display enhanced primary production from regenerated nutrients relative to the adjacent sea (1).

Finally, the authors claim that oyster farming is a scalable mCDR option, but they provide no evidence to support this. This is particularly important given that they advocate low-density aquaculture in areas that are often spatially restricted and heavily used.
